# Correction: Dynamic landscape of protein occupancy across the *Escherichia coli* chromosome

**DOI:** 10.1371/journal.pbio.3001557

**Published:** 2022-04-27

**Authors:** Lydia Freddolino, Haley M. Amemiya, Thomas J. Goss, Saeed Tavazoie

In the first sentence of subsection IPOD-HR interface extraction in the Methods section, the centrifugation force is incorrectly listed as 169,000 x g. The correct sentence is: Prior to interface extraction, samples were clarified by centrifugation for 10 minutes at 15,900 x g at 4 C.
