# Supplementary material for: Dynamic landscape of protein occupancy across the Escherichia coli chromosome
Source: PLoS Biol. 2021 Jun 25;19(6):e3001306. doi: 10.1371/journal.pbio.3001306 (PMC8282354; doi:10.1371/journal.pbio.3001306)
Supplement: S1 Text — See also S5–S8 Figs. (PDF) [file pbio.3001306.s001.pdf]

S1 Text: Effects of RNA polymerase on large-scale protein occupancy

In the IPOD-HR method, we address the contribution of RNA polymerase occupancy to the overall protein occupancy signal through two steps: first, immediately prior to crosslinking, cells are treated with rifampin for 10 minutes. We have calibrated the rifampin treatment to allow sufficient time for in-progress transcripts to finish while minimizing perturbation of cellular physiology (building on data from [1], as well as direct calculation of the required time based on the elongation rate of RNA polymerase and lengths of *E. coli* transcripts). As rifampin inhibits promoter clearance but not the transcriptional initiation nor completion of already-elongating transcripts [2], the result will thus be to cause an accumulation of RNA polymerase at active promoters, while clearing the majority of polymerase occupancy from gene bodies, and thus substantially simplifying the identification of RNA polymerase occupancy. In addition, all IPOD-HR experiments described here were performed in parallel with RNA polymerase ChIP-seq experiments under the same conditions, permitting calibrated subtraction of RNA polymerase occupancy from active promoters to reveal changes in regulatory protein occupancy (as schematized in **Fig 1B-C**). However, the fact that transcriptional inhibition is known to affect nucleoid condensation [3–6] prompted us to directly inspect the effects of rifampin on genome-wide protein binding, with a particular emphasis on the effects on EPOD formation and transcription factor binding.

To illustrate the effects of rifampin treatment on regions of extremely high or low RNA polymerase occupancy, we performed IPOD experiments under our baseline growth condition (WT cells undergoing exponential growth in M9/RDM/glu medium) following the same procedure as used for all other samples in the present study, but omitting the rifampin treatment (*n.b.* the data sets of [7] were obtained under a slightly different condition, during exponential growth in LB medium). An overall analysis of the resulting EPOD calls (**S5 Fig**) demonstrates that the -RIF EPOD set arising from our methods show good correlations with both the heEPOD and tsEPOD calls from [7], whereas the +RIF EPOD set from our method (used throughout the rest of text) shows a much stronger correlation with the Vora tsEPOD set and a weaker correlation with heEPODs. It is also important to note that the fraction of locations from the Vora tsEPOD set that is contained within our relaxed +RIF EPOD calls (0.89) is in line with the equivalent overlaps between the EPOD sets observed across different conditions in our main data sets (upper triangle of **Fig 6D**), further indicating that the EPODs identified via IPOD-HR closely resemble the original Vora tsEPODs.

To obtain a more detailed picture of the effects of rifampin on protein occupancy in our assays, we show an example of a highly expressed EPOD (heEPOD) from the original IPOD data sets in **S6 Fig**. In the original IPOD data set [7], strong protein occupancy was noted throughout the cluster of ribosomal protein operons running from *rplQ* to *rpsJ*. Here, we see from the rifampin-omitted (-RIF) samples that the protein occupancy profile in this region is dominated by RNA polymerase occupancy, closely matching the bounds of the originally called heEPOD. On the other hand, in the rifampin-treated samples (+RIF), the vast majority of occupancy in this region is lost, with peaks only apparent at a few points within the region of interest (likely corresponding to highly active promoters). After subtraction of the scaled RNA polymerase

occupancy to yield the IPOD-HR signal, the only prominent peak remaining in the region is in the *gspA*-*gspC* intergenic region, which has been demonstrated to be repressed by H-NS binding (although the precise binding location was previously unknown [8]).

The occupancy observed in the ribosomal protein operon cluster described above contrasts strongly with that seen in the transcriptionally silent EPODs (tsEPODs) found in [7]. In the region of the tsEPOD shown in **S7 Fig**, for example, we observe that in the -RIF samples, there is continuous protein occupancy but essentially no RNA polymerase occupancy throughout the large tsEPOD that was originally observed to span the *waaQGPSBOJYZU* operon, whereas both strong IPOD occupancy and strong RNA polymerase occupancy are apparent on a nearby heEPOD covering the *rpmBG* operon. Treatment with rifampin does not substantially alter the high level of overall protein occupancy throughout the *waa* region, whereas it restricts occupancy near *rpmBG* to active promoters only. As a result, in the ChIP-subtract IPOD-HR signal, strong occupancy remains throughout the *waaQGPSBOJYZU* operon, resulting in an EPOD call nearly identical to the original tsEPOD in that region from [7]. One feature of the +RIF samples that requires consideration is the fact that several RNA polymerase occupancy peaks appear in regions such as tsEPODs where no comparable occupancy is apparent in -RIF samples. We attribute these additional peaks to the fact that during rifampin treatments, concentrations of free RNA polymerase will rise substantially due to the immobilization of polymerase at transcription start sites; thus, occupancy at normally weak promoters through the chromosome will increase. We find, however, that the ChIP-subtraction step of our IPOD-HR data processing pipeline accurately removes RNA polymerase occupancy at both normally-active promoters and those showing RNA polymerase binding only in the presence of rifampin treatment (as seen by the well-calibrated removal of RNA polymerase occupancy in the +RIF tracks of **S6 Fig and S7 Fig**, for example). Overall, we find that even in the +RIF samples, RNA polymerase occupancy remains very well correlated with transcript levels (**Fig 3E**), can be cleanly subtracted to yield condition-appropriate changes in transcription factor occupancy (**Fig 1C**); furthermore, brief rifampin treatment does not appear to substantively alter large-scale protein occupancy other than that directly attributable to RNA polymerase binding (**S6 Fig** vs **S7 Fig**). Given that rifampin treatment permits cleaner subtraction of RNA polymerase occupancy, while not perturbing either the local or large-scale protein binding patterns that are the subject of our interest, we make use of it throughout the IPOD-HR data sets shown in the present work.

References

1. Herring CD, Raffaelle M, Allen TE, Kanin EI, Landick R, Ansari AZ, et al. Immobilization of Escherichia coli RNA polymerase and location of binding sites by use of chromatin immunoprecipitation and microarrays. J Bacteriol. 2005;187: 6166–6174.

2. Campbell EA, Korzheva N, Mustaev A, Murakami K, Nair S, Goldfarb A, et al. Structural mechanism for rifampicin inhibition of bacterial rna polymerase. Cell. 2001;104: 901–912.

3. Cabrera JE, Cagliero C, Quan S, Squires CL, Jin DJ. Active transcription of rRNA operons condenses the nucleoid in Escherichia coli: examining the effect of transcription on nucleoid structure in the absence of transertion. J Bacteriol. 2009;191: 4180–4185.

4. Pettijohn DE, Hecht R. RNA molecules bound to the folded bacterial genome stabilize DNA folds and segregate domains of supercoiling. Cold Spring Harb Symp Quant Biol. 1974;38: 31–41.

5. Cabrera JE, Jin DJ. The distribution of RNA polymerase in Escherichia coli is dynamic and sensitive to environmental cues. Mol Microbiol. 2003;50: 1493–1505.

6. Dworsky P, Schaechter M. Effect of rifampin on the structure and membrane attachment of the nucleoid of Escherichia coli. J Bacteriol. 1973;116: 1364–1374.

7. Vora T, Hottes AK, Tavazoie S. Protein occupancy landscape of a bacterial genome. Mol Cell. 2009;35: 247–253.

8. Francetic O, Belin D, Badaut C, Pugsley AP. Expression of the endogenous type II secretion pathway in Escherichia coli leads to chitinase secretion. EMBO J. 2000;19: 6697–6703.
