# Supplementary material for: Dynamic landscape of protein occupancy across the Escherichia coli chromosome
Source: PLoS Biol. 2021 Jun 25;19(6):e3001306. doi: 10.1371/journal.pbio.3001306 (PMC8282354; doi:10.1371/journal.pbio.3001306)
Supplement: S2 Text — IPOD-HR, in vivo protein occupancy display—high resolution; TFBS, transcription factor binding site. (PDF) [file pbio.3001306.s002.pdf]

S2 Text: Recovery of known TF binding site profiles from IPOD-HR data and inferred motifs

As noted in the main text, while the majority of newly inferred motifs from IPOD-HR data do not match the motif of a known transcription factor, 89% of motifs in the SwissRegulon database have significant matches to at least one of our inferred motifs (this number remains at 70% with the redundancy-pruned motif set). The opportunity thus arises to consider the extent to which complete TF regulons could be re-identified based on the inferred motifs. Several caveats must be kept in mind to even consider such an exercise: (i) while the sets of known binding sites for transcription factors have typically been obtained through a series of experiments targeting conditions specific to each factor, the present IPOD-HR datasets cover relatively few conditions and cannot represent the complete profile of occupancy changes that could be observed; (ii) because our motif inference is designed to optimize the coverage of observed occupancy peaks with a minimal, nonredundant set of motifs, there is no guarantee that a unique 1:1 mapping exists between inferred motifs and TFs, or between occupancy locations and inferred motifs; (iii) the present method for motif inference does not incorporate information about the co-variance of occupancy at different locations to assign binding locations to the same factors (although, as noted in the main text, such methods are currently under development); and (iv) it is likely that in some cases even well-studied factors have additional, as-yet-unidentified, binding sites.

With the above noted limitations in mind, it is still possible to assess the ability of our inferred motifs to recapitulate known TF regulons. For this purpose, we considered only the motifs from our non-redundant set that had TOMTOM hits ($E<0.5$) to a known motif from the SwissRegulon database, calculated the overlap of binding sites for each such inferred motif with the known binding sites for the corresponding TF (from RegulonDB), and compared that overlap to what would be obtained using predicted binding sites for the motif obtained directly from SwissRegulon. As seen in panel A of **S8 Fig**, the majority (76%) of the inferred motifs show an enrichment of overlap with known binding sites for the identified corresponding TF, with a distribution that overlaps (but is lower than) the enrichments observed for the actual SwissRegulon motifs. A similar trend is apparent when considering the recall of known sites (panel B of **S8 Fig**). Thus, while not reaching the quality of known motifs for each individual transcription factor, the set of binding sites implied by the motifs inferred from IPOD-HR nevertheless allow recovery of a large fraction of the sites for the TFs that they most closely match. We note that due to the absence of a clear set of gold standard negative binding sites, we have focused on the ability of the inferred motifs to identify known TFBSs at rates substantially higher than what would be expected by chance, but cannot explicitly calculate a precision or similar statistics.

Given the ambiguities outlined above in identifying the direct correspondences between IPOD-HR inferred motifs and individual TFs, it is also instructive to consider the extent to which the collected set of binding sites implied by IPOD-HR matches the known binding sites for the set of TFs considered here. To investigate these overlaps, we considered two sets of merged binding site predictions, one considering all IPOD-HR peaks (at a calling threshold of 4.0; see main text for details), and one considering all of the identified binding sites for our nonredundant

set of new motifs. The recall for binding sites in the regulons of known TFs is shown for both of these sets of binding site predictions in panel C of **S8 Fig**. We observe that for both IPOD-HR based binding site sets, the recall for known TFBSs is substantially higher than would be expected by chance for nearly all TFs; it is also notable that the recall for the motif-based sets is substantially higher than that obtained simply using the IPOD-HR peaks themselves, demonstrating the additional utility of the newly inferred motifs.

Taken together, our findings here indicate that the binding sites implied by our newly inferred motifs, while imperfect, can provide a substantial amount of evidence regarding the binding sites for well-characterized TFs with similar motifs. We expect that in the future the observed performance could be further improved by consideration of experimental data from a broader range of conditions, and the improved methods for motif inferences alluded to above. Nevertheless, the IPOD-HR based motifs provide an important set of draft binding sites even for well characterized TFs, and can likely play a similar role for currently uncharacterized TFs.
