# Supplementary figures and images for: Dynamic landscape of protein occupancy across the Escherichia coli chromosome
Source: PLoS Biol. 2021 Jun 25;19(6):e3001306. doi: 10.1371/journal.pbio.3001306 (PMC8282354; doi:10.1371/journal.pbio.3001306)

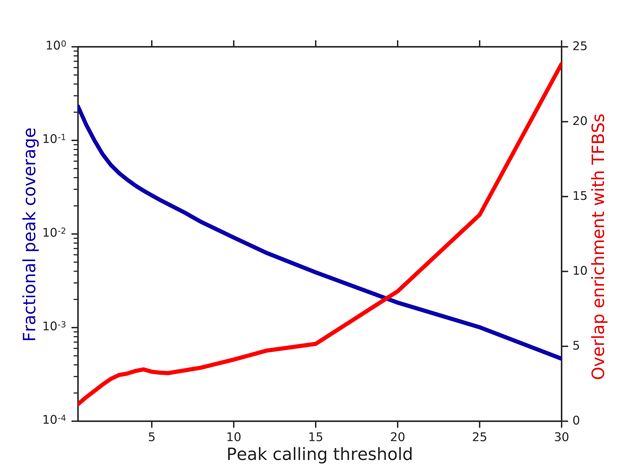

Supplement: S1 Fig — Data are shown for the WT cells in the RDM condition. Shown is the fraction of the entire genome contained in peak calls (left vertical axis, blue line) or the enrichment of TFBSs overlapping those peak calls relative to that expected by chance (right vertical axis, red line). Overlaps at all shown thresholds were statistically significant (p < 0.01, permutation test in each case). RDM, rich defined medium; TFBS, transcription factor binding site; WT, wild-type. (PNG) [file pbio.3001306.s003.png]

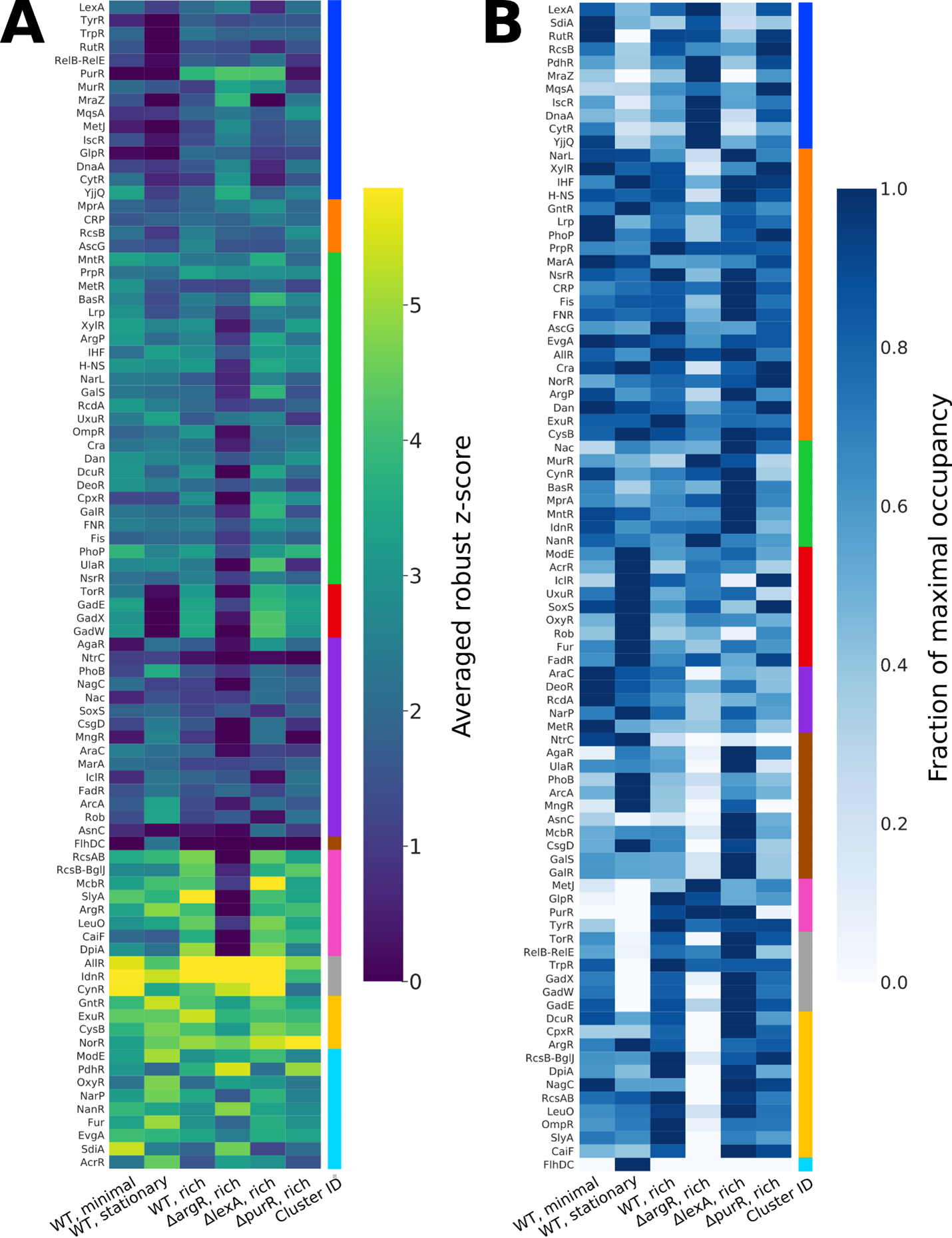

Supplement: S2 Fig — (A) Shown for each TF (row) is the geometric mean of site-level occupancies for all detectable sites for that TF under that condition. “Detectable sites” refer to RegulonDB-annotated sites which had a robust z-score of at least 3 under at least 1 condition; factors with fewer than 3 detectable sites were excluded. The values within a single site, for a single condition, are summarized by the maximum occupancy within that site, reflecting the peak of the observed binding signal. The TFs are ordered based on a consensus clustering approach as applied for Fig 3 of the main text. Raw data on the underlying site-level occupancies are given in S8 Data. (B) As in A, subsequently scaling each row by its maximum value so the highest occupancy condition for each TF receives a score of 1.0. TF, transcription factor. (PNG) [file pbio.3001306.s004.png]

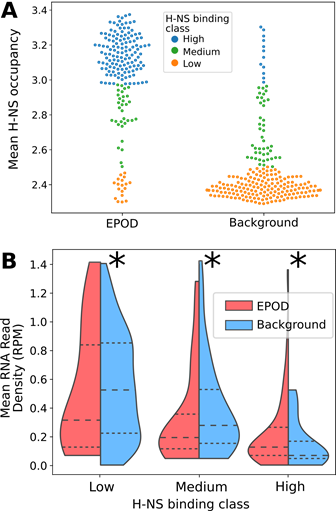

Supplement: S3 Fig — (A) Mean levels of H-NS binding (data from [44]) for all EPODs called in the WT rich media condition; each point shows either an EPOD or a single contiguous non-EPOD region. Each point is colored by its classification into high, medium, or low H-NS binding using a Gaussian mixture model with 3 groups, after removal of outliers using the local outlier factor [80] as implemented in the python scikit-learn module [81], using 25 neighbors and default settings for other parameters. (B) Distributions of mean RNA read density stratified by the H-NS binding categories shown in panel A, with each case divided by EPOD status. The median of each group is shown by a dashed line and the 25th and 75th quartiles by dotted lines. “*” indicates a significant difference between the EPOD vs. background groups (p < 0.05, Mann–Whitney U test). EPOD, extended protein occupancy domain; WT, wild-type. (PNG) [file pbio.3001306.s005.png]

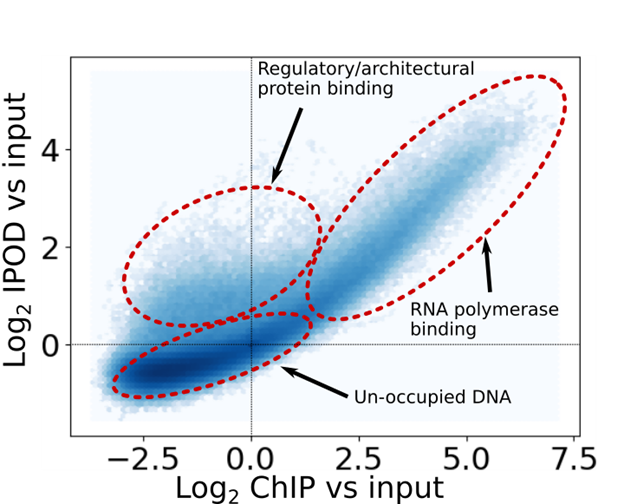

Supplement: S4 Fig — Shown is a density plot of the log2(IPOD/Input) signal vs. log2(RNA polymerase ChIP/Input) signal, demonstrating the presence of 3 subpopulations of genomic positions: unbound positions (without enrichment using either protein occupancy profiling method), RNA polymerase occupancy (part of a highly correlated region of high IPOD occupancy and high RNA polymerase occupancy), and occupancy with other proteins (which shows high IPOD occupancy but low RNA polymerase occupancy). Note that there is no corresponding population of high RNA polymerase occupancy but low IPOD occupancy, rather, the RNA polymerase-bound regions are a subset of the regions detected by IPOD. Color intensity scales logarithmically with bin occupancy. ChIP, chromatin immunoprecipitation. (PNG) [file pbio.3001306.s006.png]

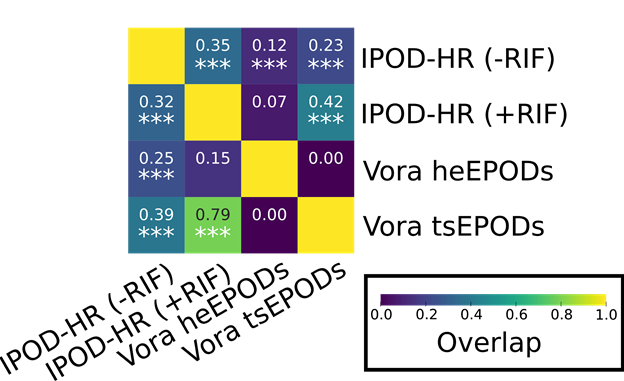

Supplement: S5 Fig — Shown in the heat map are the fraction of EPODs from the EPOD set defined by the row label that overlap the EPOD set defined by the column label. Asterisks reflect p-values arising from a Monte Carlo permutation test (1,000 random circular permutations of the EPOD locations; * p < 0.05, ** p < 0.01, *** p < 0.001). p-Values for the overlaps between the +RIF IPOD-HR EPOD set and the Vora heEPODs were >0.8 for both directions of comparisons; a full list of values is given in S2 Table. EPOD, extended protein occupancy domain; heEPOD, highly expressed extended protein occupancy domain; IPOD-HR, in vivo protein occupancy display—high resolution. (PNG) [file pbio.3001306.s007.png]

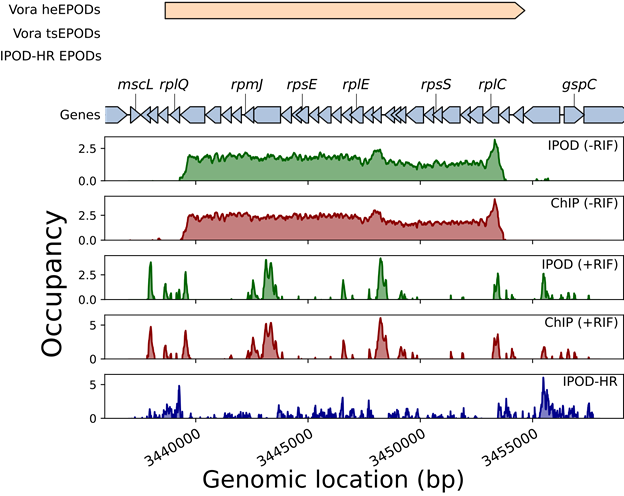

Supplement: S6 Fig — Shown are occupancy signals for interphase-extracted, RNA polymerase ChIP, and ChIP-subtracted IPOD occupancy (IPOD-HR) samples in the vicinity of a large cluster of ribosomal protein genes (running from rplQ to rpsJ). Signals are log2 extracted:input ratios (for IPOD and ChIP samples) or ChIP-subtracted robust z scores (IPOD-HR). ChIP, chromatin immunoprecipitation; IPOD-HR, in vivo protein occupancy display—high resolution. (PNG) [file pbio.3001306.s008.png]

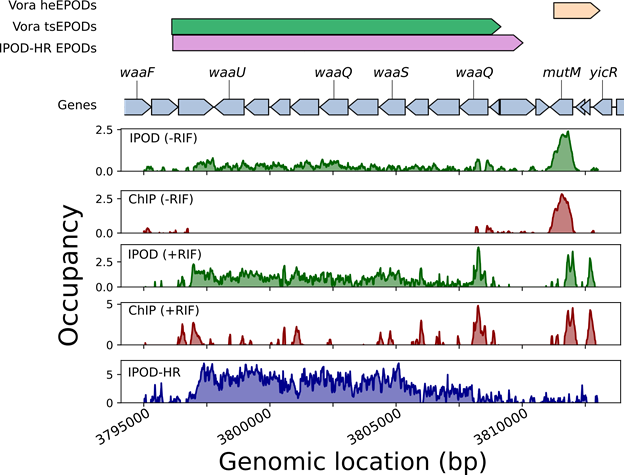

Supplement: S7 Fig — Shown are occupancy signals for interphase-extracted, RNA polymerase ChIP, and ChIP-subtracted IPOD occupancy (IPOD-HR) samples in the vicinity of the waaQGPSBOJYZU operon, which was identified as a strong tsEPOD in [10]. Signals are log2 extracted:input ratios (for IPOD and ChIP samples) or ChIP-subtracted robust z scores (IPOD-HR). ChIP, chromatin immunoprecipitation; IPOD-HR, in vivo protein occupancy display—high resolution; tsEPOD, transcriptionally silent extended protein occupancy domain. (PNG) [file pbio.3001306.s009.png]

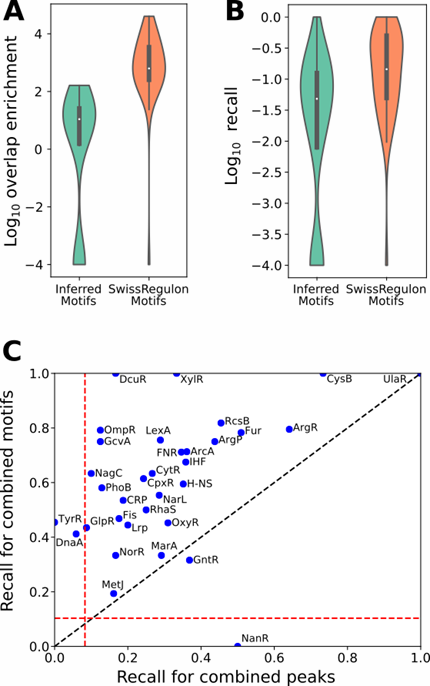

Supplement: S8 Fig — (A) Violin plots showing the log10-fold enrichment (or depletion) of overlap between the indicated motif-based binding site calls (using IPOD-HR inferred motifs or motifs from SwissRegulon) with annotated binding sites from RegulonDB; matches of inferred motifs with TFs arise from TOMTOM calls (see text for details). A pseudocount of 0.0001 is added to each overlap. For motif hits in the present figure, “loose” motif hits were used if strict hits were not available (see Methods for details). (B) Log10 recall for identification of annotated (from RegulonDB) binding sites for each set of motif-based calls indicated in panel A; a pseudocount of 0.0001 is added to each value to avoid singularities. (C) Recall of annotated sites for each indicated TF (matching those shown in panels A and B) using either the union of all peaks called from our IPOD-HR data set at a peak calling threshold of 4 (“combined peaks”) or the union of all binding sites for our nonredundant motif set (“combined motifs”). Red dashed lines show the fraction of the genome covered by the peaks and motifs (depending on the axis), and thus represent the recalls that would be expected solely by chance. IPOD-HR, in vivo protein occupancy display—high resolution; TF, transcription factor. (PNG) [file pbio.3001306.s010.png]
