# Supplementary material for: Dynamic landscape of protein occupancy across the Escherichia coli chromosome
Source: PLoS Biol. 2021 Jun 25;19(6):e3001306. doi: 10.1371/journal.pbio.3001306 (PMC8282354; doi:10.1371/journal.pbio.3001306)
Supplement: S1 Table — The higher means and rightward skews of all other data sets relative to the “Coding, No TF” portion indicates that the portions of the genome with higher IPOD-HR robust z-scores are associated with noncoding regions and annotated TFBSs. The “vs. Coding No TF” column gives the p-value for a permutation test comparing the mean z-scores in the indicated genomic region with those in the “Coding, No TF” region; the permutation test was conducted using 200 random rotations of the data values relative to the feature coordinates, ensuring that the correlation structures of both data and features were conserved. IPOD-HR, in vivo protein occupancy display—high resolution; TF, transcription factor; TFBS, transcription factor binding site; WT, wild-type. (PDF) [file pbio.3001306.s011.pdf]

Genomic Region	Mean	Standard deviation	Skew	vs. Coding No TF
Noncoding, No TF	0.80	2.08	0.38	<0.005
Noncoding, TF site	1.06	2.31	0.65	<0.005
Coding, No TF	0.17	1.26	-0.03	--
Coding, TF Site	0.34	1.69	0.20	0.075
(Standard normal)	0.00	1.00	0.00	--
