# Supplementary material for: Dynamic landscape of protein occupancy across the Escherichia coli chromosome
Source: PLoS Biol. 2021 Jun 25;19(6):e3001306. doi: 10.1371/journal.pbio.3001306 (PMC8282354; doi:10.1371/journal.pbio.3001306)
Supplement: S3 Table — (PDF) [file pbio.3001306.s013.pdf]

Identified protein	Accession Number	Molecular Weight	Total Spectrum Count
UlaR	tr|C3SFV2|C3SFV2_ECOLX	28 kDa	16
YieP	tr|E2QHU3|E2QHU3_ECOLX	26 kDa	9
RpsC	tr|C3SQX2|C3SQX2_ECOLX	26 kDa	7
RpoC	tr|C3SIA2|C3SIA2_ECOLX	155 kDa	11
RpoB	tr|E2QJ13|E2QJ13_ECOLX	151 kDa	7
RuvA	tr|C3T5R2|C3T5R2_ECOLX	22 kDa	10
FliA	tr|C3SDE6|C3SDE6_ECOLX	28 kDa	6
RpoA	tr|C3SR67|C3SR67_ECOLX	37 kDa	7
RpsD	tr|C3SR62|C3SR62_ECOLX	23 kDa	3
RdgC	tr|E2QGD8|E2QGD8_ECOLX	34 kDa	3
UvrA	tr|C3SHF7|C3SHF7_ECOLX	104 kDa	5
CysB	tr|C3TC57|C3TC57_ECOLX	36 kDa	5
FabR	tr|E2QIZ8|E2QIZ8_ECOLX	24 kDa	4
GroL	tr|Q548M1|Q548M1_ECOLX	57 kDa	3
IhfB	tr|Q14F22|Q14F22_ECOLX	11 kDa	4
RplC	tr|C3SQU2|C3SQU2_ECOLX	22 kDa	2
AccB	tr|C3SRL7|C3SRL7_ECOLX	17 kDa	4
AmiA	tr|E2QPR8|E2QPR8_ECOLX	31 kDa	3
IhfA	tr|Q14F23|Q14F23_ECOLX	11 kDa	2
Ppx	tr|C3T027|C3T027_ECOLX	58 kDa	2
RplB	tr|C3SQV7|C3SQV7_ECOLX	30 kDa	2
RpoE	tr|Q0P6M2|Q0P6M2_ECOLX	22 kDa	2
FabZ	tr|C3TPH7|C3TPH7_ECOLX	17 kDa	3
PolA	tr|E2QI51|E2QI51_ECOLX	103 kDa	2
TufB	tr|E2QFJ4|E2QFJ4_ECOLX (+1)	43 kDa	2
