# Supplementary material for: Dynamic landscape of protein occupancy across the Escherichia coli chromosome
Source: PLoS Biol. 2021 Jun 25;19(6):e3001306. doi: 10.1371/journal.pbio.3001306 (PMC8282354; doi:10.1371/journal.pbio.3001306)
Supplement: S4 Table — The “Median difference” column refers to the difference in median robust Z-scores between EPODs and all other sites in the genome, with positive values indicating higher levels within EPODs. p-Values for a significant difference are obtained using a resampling test, with 1,000 random circular permutations of the EPOD locations on the genome (thus preserving the correlation structure of genomic features); q-values are obtained by correction of the p-values using the Benjamini–Hochberg method [82]. All sequence features were subjected to a 500-bp rolling mean prior to overlap calculation. EPOD, extended protein occupancy domain. (PDF) [file pbio.3001306.s014.pdf]

Feature	Median difference	p value	q value	Reference
Transcriptional propensity	-1.009	0.001	0.001	[1]
Supercoiling density	-0.256	0.487	0.487	[2]
Normalized Tn5 integration density	4.605	0.001	0.001	[1]
Fis binding	-1.060	0.001	0.001	[3]
H-NS binding	12.068	0.001	0.001	[3]
HU binding	-0.289	0.042	0.048	[4]
LRP binding	0.462	0.091	0.097	[5]
SeqA binding	-0.255	0.003	0.004	[6]
Dam sites	-0.674	0.001	0.001	Sequence analysis
AT content	1.855	0.001	0.001	Sequence analysis
RNA	-0.682	0.001	0.001	Present study
MGW	-0.084	0.001	0.001	Calculated using DNAshapeR [7]
HelT	0.071	0.001	0.001	
ProT	-0.169	0.001	0.001	
Roll	-0.086	0.001	0.001	

References

1. Scholz SA, Diao R, Wolfe MB, Fivenson EM, Lin XN, Freddolino PL. High-Resolution Mapping of the Escherichia coli Chromosome Reveals Positions of High and Low Transcription. Cell Syst. 2019;8: 212–225.e9.
2. Lal A, Dhar A, Trostel A, Kouzine F, Seshasayee ASN, Adhya S. Genome scale patterns of supercoiling in a bacterial chromosome. Nat Commun. 2016;7: 11055.
3. Kahramanoglou C, Seshasayee ASN, Prieto AI, Ibberson D, Schmidt S, Zimmermann J, et al. Direct and indirect effects of H-NS and Fis on global gene expression control in Escherichia coli. Nucleic Acids Research. 2011. pp. 2073–2091. doi:10.1093/nar/gkq934
4. Prieto AI, Kahramanoglou C, Ali RM, Fraser GM, Seshasayee ASN, Luscombe NM.

Genomic analysis of DNA binding and gene regulation by homologous nucleoid-associated proteins IHF and HU in Escherichia coli K12. Nucleic Acids Res. 2012;40: 3524–3537.

5. Kroner GM, Wolfe MB, Freddolino PL. Escherichia coli Lrp regulates one-third of the genome via direct, cooperative, and indirect routes. J Bacteriol. 2018. doi:10.1128/JB.00411-18
6. Joshi MC, Magnan D, Montminy TP, Lies M, Stepankiw N, Bates D. Regulation of sister chromosome cohesion by the replication fork tracking protein SeqA. PLoS Genet. 2013;9: e1003673.
7. Chiu T-P, Comoglio F, Zhou T, Yang L, Paro R, Rohs R. DNAshapeR: an R/Bioconductor package for DNA shape prediction and feature encoding. Bioinformatics. 2016;32: 1211–1213.
