# Supplementary material for: Dynamic landscape of protein occupancy across the Escherichia coli chromosome
Source: PLoS Biol. 2021 Jun 25;19(6):e3001306. doi: 10.1371/journal.pbio.3001306 (PMC8282354; doi:10.1371/journal.pbio.3001306)
Supplement: Attachment — Submitted filename: 109625_1_rebuttal_1894643_qgk6bc.docx [file pbio.3001306.s024.docx]

**High-level outline of proposed revisions based on first round of review**

*Freddolino et al. Dynamic landscape of protein occupancy across the Escherichia coli chromosome (RC-2020-00418)*

We are grateful to the reviewers for their thorough reading of our work, their enthusiastic comments, and many useful suggestions for strengthening the paper further. Overall, the reviewers appear to agree that the method appears broadly useful and will help to drive the field forward; key overall significance statements include:

*“The key conclusions are convincing. Of course the methodology has inbuilt issues, like all top-down methods that rely on a particular probe, and these will bias the results, but the authors assess the pros- and cons realistically.”* (Reviewer 1)

*“This will be a valuable addition to the existing toolkit of high-throughput methods and has a great potential when applied to other organisms and physiological conditions.”* (Reviewer 2)

*“The authors have done an excellent job showing, with specific examples, potential applications of their new technology.”* (Reviewer 3)

Based on both these top-level summary comments, and the totality of the reviewer comments, there appears to be a consensus that our paper both provides an important technical advance (in the form of the IPOD-HR method), and contains significant biological insights and observations that will have a profound effect on ongoing work in the field. Both our ability to contribute to ongoing efforts to comprehensively map bacterial transcriptional regulatory networks, and our findings regarding the importance and nature of several different classes of heterochromatin-like extended protein occupancy domains (EPODs) on the apparent suppression of parasitic genetic elements, will be of broad interest. In addition, as recognized by the reviewers, the methods described here are ripe for application to other physiological conditions and bacterial species; indeed, several such studies are already in progress.

At the same time, the reviewers (particularly Reviewer 3) provide several excellent suggestions that we agree can strengthen the paper further. We plan the following additions in order to address the reviewer comments and allow the paper to reach its full potential:

In response to Reviewer #1’s desire for additional examples of the IPOD-HR occupancy, we already provide flat files containing all of the occupancy traces in a flat format, and will further emphasize this availability and provide additional files compatible with all major genome browsers. We will also provide additional specific examples of how IPOD-HR occupancy traces look particularly in regions with many well-characterized binding sites.

Reviewers #2 and #3 had several methodological questions which we will address with more detailed commentary on the analysis algorithms used (and sensitivity analysis of key parameters that we employ), as well as complete release of the computational tools that we use under an open source license.

Reviewer #2 also requested additional functional validation of the YieP binding sites that we identified; we will perform these experiments using a lacZ reporter system as they suggest.

Reviewer #3 had several excellent suggestions regarding the integration and cross referencing of our findings with existing data, particularly that in RegulonDB. Indeed, we had already performed several of the requested analyses (e.g., tracking average condition-dependent occupancy of all sites for both known and newly called TF binding motifs), but had omitted some of that information for the sake of brevity. Given the obvious interest, we intend to implement essentially all of Reviewer #3’s suggested additional calculations (e.g., the aforementioned occupancy tracking, testing for regulon overlaps, etc.), with the exception of a few cases where there is simply not enough data available at present to implement what is being recommended.

We expect that with the modifications and additions outlined above (which we are already proceeding with, and which we expect will take an additional 4-6 weeks to complete), this manuscript will provide an important contribution to the field, and will be of interest to the broad readership of *The EMBO Journal.* A fully detailed Response to Reviewers would be included with the revised manuscript.
