# Supplementary material for: Dynamic landscape of protein occupancy across the Escherichia coli chromosome
Source: PLoS Biol. 2021 Jun 25;19(6):e3001306. doi: 10.1371/journal.pbio.3001306 (PMC8282354; doi:10.1371/journal.pbio.3001306)
Supplement: Attachment — Submitted filename: response_to_reviewers_final.docx [file pbio.3001306.s026.docx]

**Response to Reviewers**

We are grateful to the reviewers for their thorough reading of our work, their enthusiastic comments, and many useful suggestions for strengthening the paper further. Overall, the reviewers appear to agree that the method appears broadly useful and will help to drive the field forward.

Based on the totality of the reviewer comments, there appears to be a consensus that our paper both provides an important technical advance (in the form of the IPOD-HR method), and contains specific data and biological insights that will have a profound effect on ongoing work in the field. Both our ability to contribute to ongoing efforts to comprehensively map bacterial transcriptional regulatory networks, and our findings regarding the importance and nature of several different classes of heterochromatin-like extended protein occupancy domains (EPODs) on the apparent suppression of parasitic genetic elements, will be of broad interest. In addition, as recognized by the reviewers, the methods described here are ripe for application to other physiological conditions and bacterial species; indeed, several such studies are already in progress.

At the same time, the reviewers (particularly Reviewer 3) provide several excellent suggestions that we have implemented to strengthen the paper further. Here we summarize the most substantial changes that have been made in order to allow the paper to reach its full potential:

- In response to the comments of reviewer #2 that the LOESS method for ChIP signal subtraction seemed potentially arbitrary, we carefully considered alternative, simpler methods. We developed a new approach based on a linear fit that is simpler, has guaranteed properties regarding removal of high levels of RNA polymerase occupancy, requires fewer parameters and no downsampling, and ultimately appears qualitatively more effective at removing signal due to RNA polymerase occupancy. We have reanalyzed all data present in the paper using the new approach, which results in minor changes to virtually all figures, but does not affect our conclusions. We thank the reviewer for their suggestions, and believe that we are now able to provide an even cleaner data set and stronger foundation for future applications of the method.
- We have provided many additional specific examples of IPOD-HR occupancy traces at regions of interest, and distributed several additional genome browser-friendly supplementary data files to allow researchers to view and inspect the data.
- We have substantially expanded the description of the computational methods, and now also distribute our analysis pipeline both from a public github repository, and in a singularity container to facilitate reproducible performance of the analysis.
- We performed reporter experiments demonstrating that YieP acts directly as a transcriptional activator at the *sdaC* promoter.
- We have added extensive additional analysis of our newly inferred motifs and cross-referencing with existing databases such as RegulonDB, in order to provide additional insight into likely functional roles played by these motifs.

We believe that with the additions that we have now made, this manuscript will provide a strong contribution to the field, and will be of interest to the broad readership of *PLoS Biology.* Point by point responses to the comments of the reviewers are included below.

***Reviewer #1 (Evidence, reproducibility and clarity (Required)):***

***Comment:***

*The paper describes a technique that produces an IPOD (in vivo protein occupancy display) across the entire E coli chromosome. By subtracting out signals due to RNA polymerase, this display is HR (high resolution) in the sense that the authors can see signals due to individually bound proteins. The drawback of the method is that the signals seen in the IPOD are anonymous, but the authors describe in some detail how the display can be exploited. The paper is clearly written and presented, and will be accessible to students, with some effort, and easy to grasp for those who have followed the topic, especially those familiar with the previous 2009 Mol Cell Vora et al paper.*

*The key conclusions are convincing. Of course the methodology has inbuilt issues, like all top-down methods that rely on a particular probe, and these will bias the results, but the authors assess the pros- and cons realistically. Concerning the data, I would have liked to see more of the IPOD in the format in Figure 2. I guess this is somewhere in the Supplementary data, but I was unable easily to access it. What is really needed is a user-friendly accessible browser format.*

**Response**

We now provide an additional supplementary file, **Supplementary Data 1**, that contains genome browser-ready bedgraph files with the occupancy traces that are displayed in the text, so that users can readily inspect any region of interest. We highlight the availability of these files in the text:

To facilitate inspection of other regions of interest, we provide complete occupancy traces for the conditions studied in Supplementary Data 1.

Additional snapshots are also provided in response to the following reviewer comment.

***Comment:***

*The authors make a convincing case that certain 'spikes' in the IPOD correspond to individual proteins bound. So what about promoter regions where several proteins are bound e.g. the lac operon regulatory region where one might expect to see Lac Repressor and CAP? Or the galETK promoter where there could be Gal Repressor, HU and CAP? Readers might like to check their favorite promoters for themselves. At the very least, the authors need to make a clear statement about what is missing.*

**Response:**

The reviewer makes several excellent points here. To address the question regarding the appearance of IPOD-HR data at ‘crowded’ promoters with many nearby (and even overlapping) binding sites, we show examples of two such promoters in the new **Fig. 3F-G** and accompanying text (paragraph beginning “In addition to revealing the occupancy of individual…”). As we show there, while the occupancy landscape is obviously more complex in the presence of many nearby binding sites, the resolution of IPOD-HR is sufficient to resolve nearby sites as long as they are not truly overlapping. We also present heuristics for assigning the most likely factor giving rise to occupancy even when sites do overlap; the formalization of these methods into a peak assignment algorithm is a topic of ongoing research. (As an aside, we focus on promoters responding to changing nitrogen source availability rather than other sugar carbon sources since all conditions presented here are in defined media with glucose as a carbon source).

Regarding the second point of indicating what is missing, we now provide a detailed description of the fraction of TFs detectable under our conditions, and possible reasons for the absence of factors that were not identified, in the paragraph beginning “Similar insights can be obtained across the *E. coli* transcriptional regulatory network…”. We do not reproduce the additions here due to their length.

***Comment:***

*For me the highlight was the observation that EPODs (extended protein occupancy domains) don't change much according to growth conditions. This leads to a very nice view of proteins on the E coli 'landscape' where regulatory features vary but EPODs don't (well, not much, anyway). The non-HNS EPODS are intriguing. Is it possible to do a subtraction of H-NS ChIP signals as was done for RNAP ChIP signals?*

**Response:**

We appreciate the reviewer’s interest in this point, and agree that this represents a fascinating line of investigation. In principle it would be possible to subtract a publicly available H-NS ChIP dataset from our occupancy profiles, and indeed we took a similar approach in the identification of non-H-NS EPODs in Figure S3. However, the only way to achieve a precision of H-NS subtraction similar to what we obtain in our RNAP subtraction would be to perform the H-NS ChIPs directly in parallel with the other experiments. As an alternative approach, we have currently ongoing work in which we are systematically deleting individual NAPs and pairs of NAPs in order to better map out the locations and identities of non-H-NS EPODs. However, these studies represent a substantial amount of additional work which we believe is beyond the scope of the present manuscript.

*Reviewer #1 (Significance (Required)):*

***Comment:***

*The opening paragraph of the discussion explains the relative merits of bottom-up versus top-down approaches to understanding bacterial regulatory networks, and carefully explains that IPOD-HR is an addition to the top-down told. This work is of interest to those concerned with bacterial chromosome organisation and the regulation of bacterial gene expression.*

**Response:**

We thank the reviewer for the suggestions and for their overall favorable evaluation of our work.

***Reviewer #2 (Evidence, reproducibility and clarity (Required)):***

*see below*

*Reviewer #2 (Significance (Required)):*

***Comment:***

*The authors describe a novel approach that allows characterization of the protein-DNA interaction dynamics at the global scale. The method is a further advance on protein occupancy display (IPOD) developed earlier by the same authors. The crucial modification introduced in the present work is the use of rifampicin treatment to distinguish the contribution from sequence-specific transcription factors and RNA polymerase. The new method is referred to as IPOD-HR (for high resolution).*

*They apply this method across different physiological conditions (exponential and stationary phase, rich or minimal medium) and validate the results using a set of well characterized transcriptional regulators (ArgR, LexA, PurR) and corresponding mutants. They then utilize an unsupervised clustering algorithm on the protein occupancy data for annotated transcription factors and show that transcription regulators belonging to the same GO term tend to cluster together across the conditions.*

*As a large number of protein occupancy peaks obtained in this study does not correspond to the annotated transcription factor binding sites in promoter, the authors select one such site and use it to fish out and identify the interacting protein (YieP). They validate the functional importance of this interaction by in vitro DNA-binding assays and RNA-Seq data for the corresponding mutant which show a significant reduction of the regulated transcript (sdaC) level.*

*Building on this result the authors examine the sequences corresponding to IPOD-HR peaks and find that about a half of them do not match any known (or predicted) transcription factor binding sites. They use computational motif discovery algorithm FIRE to IPOD-HR data to find about 200 novel motifs. These account for almost a third of the observed protein occupancy peaks. Importantly, the inferred motifs show significant enrichment in promoter but not ORF regions.*

*The last part of this work explores so-called extended protein occupancy domains (EPODs) which were first reported by the authors in the previous work and apparently correspond to kilobase-scale regions of high level of protein occupancy. The correction for RNA polymerase occupancy levels introduced in the present study allowed the authors to claim that most EPODs are transcriptionally silent, akin to eukaryotic heterochromatin regions. One striking result is that EPODs appear to be almost invariant across the range of physiological conditions used in this study. The pathways enriched in EPODs regions (prophage genes and mobile elements) as well as the strong overlap with H-NS ChIP-Seq data prompt the authors to suggest that the main role of EPODs is the transcriptional silencing of xenogenetic material. Nonetheless, a significant fraction of EPODs does not overlap with H-NS binding and provide an intriguing opportunity for further study.*

*The strength of the method is that no prior knowledge of the DNA interacting protein is required and it can be used to survey the protein occupancy of both transcription regulatory sites and large-scale protein-bound domains of bacterial chromosome. This will be a valuable addition to the existing toolkit of high-throughput methods and has a great potential when applied to other organisms and physiological conditions.*

**Response:**

We appreciate the reviewer’s careful reading, extensive summary, and positive overall evaluation.

***Comment:***

*However, several points need to be addressed before the work can be published.*

*1.My main concern is that being heavily dependent on computational analysis this study does not provide the source code for the analysis performed, specifically protein occupancy and feature (peak) calling and transcription factor co-clustering. While I agree that the inclusion of code for routine steps in NGS data processing such as QC and alignment adds little value to the reader, these are computational steps on which the entire method is built and they should be included in the manuscript as a link to the source code.*

**Response:**

We appreciate the reviewer’s comments and completely agree with the need for full source code release; indeed, we had already planned to provide this along with the manuscript. As we now note in the Methods section, the relevant source code can now be downloaded from <https://github.com/freddolino-lab/ipod_v1_2020>, including both the initial parsing of reads to occupancy data, and the key postprocessing steps noted by the reviewer. In addition, we provide both a Singularity container allowing users to apply our analysis pipeline without the need to directly replicate our development environment, and an example dataset that shows a template for analysis and allows testing of the functionality of a user environment against gold-standard data. Links to the source code are now provided in the “Analysis of NGS data” section of the Methods.

***Comment:***

*2.The description of protein occupancy and peak calling in the method section is difficult to parse and probably should be re-written for better clarity. Specific questions that arise while reading this part:*

*- Why is spline used for smoothing abundances? The choice of smoothing function and its parameters needs better explanation. The same goes for the choice of LOESS fit for RNA polymerase normalization.*

*- Where did 1.1 scaling factor come from?*

*- What's the effect of data downsampling on the fit robustness?*

*- Similarly, the selection of parameters used in peak calling in both CWT and EPODs cases needs to be explained. They seem arbitrary without additional clarification.*

**Response:**

We appreciate the reviewer’s very useful feedback, and have taken it to heart by extensively rewriting the computational methods section to enhance clarity and provide clearer justifications for the choice of any tunable parameters used in the analysis. We believe that the combination of this improved Methods text, plus the well documented source code distribution described above, will substantially enhance the ability of other researchers to understand and re-use our analysis tools.

On the particular point of the potential arbitrariness of parameter choices, we would like to point out that to some extent arbitrary cutoffs and parameter choices are an inescapable aspect of biomedical and bioinformatic research -- for example, “p<0.05”, “95% confidence intervals”, etc. represent agreed-upon compromises in the field, but are by their nature arbitrary thresholds, and what is more important in their utility is that researchers (hopefully) think carefully about the thresholds used in their own work, and at least understand what is meant by the choice of any particular threshold.

In the case of the parameters used in our analysis, they have generally been chosen on the basis of exploratory data analysis on a subset of the data because they appeared to provide a faithful representation of the data (for parameters early in the processing pipeline) or because they provide a useful threshold (in the case of later parameters such as the peak calling thresholds). In the latter case, we can say that our thresholds are, at least, as justified as (for example) a particular p-value cutoff, because we show documentation of the effects of the range of possible parameter choices on our inference. Below we provide explanatory notes and/or direct quotes from the revised manuscript addressing each of the specific points raised by the reviewer:

*Splines for abundance smoothing*. We provide the following expanded justification for the use of spline fits and abundance normalization. We also note that in general, smoothing (not interpolating) splines provide excellent performance in providing a low-pass filter on the data when the number of knots is much less than the number of data points, as was the case here.

In order to account for the higher amount of genomic DNA present near the origin of replication relative to the terminus, we fitted a smoothing spline with four evenly spaced knots and periodic boundary conditions to the input sample for each condition; the smoothing spline provides a low-pass filter on abundances that accounts for large-scale variations in DNA abundance across the genome. The use of four knots allows inflection points at the origin, terminus, and the halfway points between them.

*LOESS smoothing for ChIP subtraction*: While we believe that the LOESS smoother was an appropriate choice for estimating the contribution of the IPOD signal due to RNA polymerase occupancy, we were inspired by the reviewer’s comments to seek a simpler model with easier to interpret statistical properties. We developed a modified procedure for RNA polymerase subtraction based on fitting a linear model, specifically chosen such that the fit of IPOD as a function of RNA polymerase ChIP signal will remove 95% of the observed occupancy at the highest occupancy CHIP sites (which are interpreted to consist entirely of RNA polymerase occupancy, consistent with Figure S4). We have re-analyzed all data now presented in the paper based on this improved method for ChIP subtraction, which required fewer parameters, is easier to interpret, and based on our qualitative analysis is superior in terms of removing RNA polymerase signal. The new method is described in detail in the second paragraph of the “ DNA Sequencing and Protein Occupancy Calling” section of the Methods, which we do not reproduce here due to its length.

*1.1 scaling factor on ChIP subtraction.* The factor of 1.1 used to scale the LOESS signal during ChIP subtraction is now a moot point, as it is no longer used. However, the choice of what fraction of high-ChIP sites should be contained under the linear fit to the IPOD vs. ChIP curve remains a free parameter in our analysis – while we have chosen a value of 95% in our current method (that is, 95% of high-ChIP sites will have their occupancy completely eliminated by the subtraction, whereas the remainder may have a small amount of residual occupancy), making some choice of how conservative to be in terms of RNA polymerase subtraction is (as with many decisions such as statistical thresholds) unavoidable, but in this case we can make a clear statement about what the subtraction means. We have chosen a high value for this parameter in order to err on the side of unduly removing measured occupancy; while in principle other values could be used, in practice we believe that our results (even more so with the inclusion of comparisons with external data sets such as in the new Fig. 3B) justify the utility of the present choice, which is grounded in statistical convention. We provide a more detailed explanation of the tradeoffs involved in this parameter in the revised Methods section:

We note that the choice of slope to completely eliminate all occupancy at 95% of the high-ChIP sites represents a highly conservative choice (in terms of removing virtually all occupancy from the IPOD signal that could potentially be attributed to RNA polymerase). Any choice for this parameter must necessarily reflect a balance of risks, where lower values (close to 50%, which would roughly match a direct linear fit to the IPOD vs ChIP data points) increase the odds of identifying spurious protein occupancy peaks actually attributable to RNA polymerase, and higher values (up to 100% would be conceivably justifiable) risk erasing true regulatory protein occupancy due to overzealous RNA polymerase occupancy removal.

*Effect of downsampling on LOESS regression robustness.* The question is now moot, as no downsampling is performed.

*Selection of CWT peak calling parameters.* We now provide an expanded justification of the CWT peak calling parameters, included below. We note, in particular, that of the two potential parameters that might be considered ‘arbitrary’ here, the width range and the signal-to-noise threshold, several values of the width range are required input for the CWT algorithm (and as we now note, the values used were motivated by the fragment size distributions of our libraries). We used several different peak calling thresholds to reflect a range of stringencies, and as we note here, we report the data (and peak locations) for all of them to give the richest possible description of the data:

To identify peaks in the robust z-scaled IPOD-HR data, we applied continuous wavelet-transform (CWT) peak calling [[62]](https://paperpile.com/c/NGxbzd/GKCAM) (as implemented in the scipy.signal package), with a range of widths from 25 bp to 125 bp (at 5 bp increments, reflecting the expected range of peak sizes given the fragmentation of our input DNA) used to generate the CWT matrix, and refer to peaks based on the minimum signal-to-noise ratio threshold at which they appear as peak calls (note that in the CWT method, a range of widths must be specified as part of the algorithm, and gives rise only to a single peak set). To provide quantitative measures of the evidence for different peak calls, separate peak sets were generated at several signal to noise thresholds (a full listing can be observed in the set of thresholds shown in **Supplementary Data S2).** Each peak call was padded by 30 bp on each side to define the peak region used in subsequent analysis. We show the performance of a variety of calling thresholds in identifying known TFBSs in **Figure S1**, and based on those findings, use a threshold of 4 for all other quantitative analysis presented here. However, peak sets at all thresholds are supplied in **Supplementary Data S2**.

*Selection of EPOD calling parameters.* As we now elaborate in the revised methods section, the lower EPOD calling threshold was chosen for compatibility with earlier work (Vora 2009); the higher threshold simply reflects an increased stringency (one might say, like using p=0.005 instead of p=0.05). In practice, the concordance of the newly called strict EPOD set with those of Vora 2009, in terms of both direct overlaps and gene functionality, appears to justify the choice.

***Comment:***

*3.The functional validation of YieP binding site is not convincing enough. The transcript level depletion in mutant RNA-Seq data might be an indirect effect. A reporter gene assay will provide a more direct evidence that the site is indeed a functional transcriptional regulator.*

**Response:**

We would like to point out that from the standpoint of utility of IPOD-HR in discovering transcription factor binding sites, the question here may seem moot given the strong ChIP-exo evidence in favor of YieP binding at these positions. Nevertheless, to directly assess the potential regulatory role of these sites, we performed a series of reporter experiments in which a *lacZ* reporter was put under control of either the native *sdaC* promoter, or the same promoter with one or both putative YieP sites scrambled (see the newly added **Figure 4E**). Most notably, we find that the reporter activity with the native *sdaC* promoter but deletion of *yieP* is virtually identical to the reporter activity with the *sdaC* promoter lacking both YieP sites, but with *yieP* itself intact; both cases show significant loss of reporter activity relative to the WT cells with *sdaC* promoter. As we discuss in the text, there is additional evidence that YieP acts indirectly at the *sdaC* promoter in addition to the direct effects, as there is sign epistasis between the *yieP* genotype and status of YieP sites in the *sdaC* promoter.

***Comment:***

*4.Perhaps outside of the scope of this study but it would be great to provide some kind of functional validation to the novel transcription factor motifs reported here.*

**Response:**

We appreciate the reviewer’s interest on this point and agree that functional testing of the new regulatory motif calls would be a fascinating area of future research. However, we believe that in light of the extensive testing and cross referencing of our data with existing knowledge of TFBSs and large-scale nucleoid associated protein occupancy, as well as our specific testing of knockouts of characterized TFs, the addition of functional testing of new motifs is (as the reviewer indeed suggests) beyond the scope of the present work.

***Reviewer #3 (Evidence, reproducibility and clarity (Required)):***

***Summary:***

*In this manuscript, Freddolino and collaborators introduced their improved in vivo protein occupancy display technology (IPOD-HR) for genome-wide profiling of protein occupancy. The IPOD-HR workflow integrates protein occupancy data and RNA polymerase ChIP-seq data to identify all regions of a bacterial genome occupied by DNA-binding proteins. Using the IPOD-HR technology, the authors identified ~19,000 potential TF-DNA interactions in the genome of Escherichia coli across six different conditions/genotypes. Remarkably, protein occupancy profiles generated using IPOD-HR recapitulated a high proportion of previously reported TF binding sites in E. coli chromosome, identified ~90% of the known DNA binding motifs for E. coli TFs, and discovered thousands of novel TF binding sites that await further characterization. The authors showed that protein occupancy profiles (generated using IPOD-HR) in combination with prior knowledge about a transcriptional regulatory network (TRN) of interest may offer valuable information about the regulatory activity of TFs, uncharacterized gene promoters, and binding affinity of uncharacterized putative TFs. Therefore, this new technology may facilitate the reconstruction of condition-specific bacterial TRNs.*

**Response:**

We appreciate the reviewer’s careful reading and summary of our work.

***Major comments:***

***Comment:***

*The authors have done an excellent job showing, with specific examples, potential applications of their new technology. However, the manuscript would deeply benefit from a more comprehensive analysis of the functional implications of the TF-DNA binding events discovered using IPOD-HR. Authors should use the protein occupancy profiles they have already generated to reconstruct the binding profiles of individual regulators across different conditions (at least for global regulators with dozens of targets).*

**Response:**

We appreciate the author’s excellent suggestions for enhancing the utility of the manuscript and its connections to existing data. Because IPOD-HR does not directly reveal the identity of the DNA-binding protein giving rise to each occupancy peak, but rather, those identities must be inferred based on the sequence and other prior knowledge of the site itself, we approach these questions from a direction centered on the transcription factors themselves.

First, in order to provide a global view of the measured occupancies of sites for different TFs across conditions, we provide in **Fig. S2** the observed condition-dependent average occupancies of all TFs with at least three detectable annotated sites. Here we observe several clusters of co-acting TFs, including clear responses to nutrient deprivation and acid stress, consistent with the clustering observed in **Fig. 3C**. In the process, we also observe the important point (noted in the text) that the intensity of signal given by different TFs varies dramatically, likely due to differences in TF-DNA crosslinking efficiencies and the efficacy with which each TF partitions to the aqueous phase in our interphase extraction; thus, the normalized occupancy profiles in **Fig. S2B** are much more informative.

In order to reconstruct the binding profile for a global regulator, as suggested, we selected the case of the Lrp regulon, as we have recent ChIP-seq data for Lrp under conditions similar to those employed in our IPOD-HR experiments. We would expect *a priori* that the IPOD-HR occupancy profile across conditions at known Lrp sites to be correlated with Lrp ChIP-seq occupancy under the same conditions, although the correlation should by no means be expected to be perfect, both because binding of other factors may overlap Lrp sites, and because changes in binding of non-Lrp factors may impact the normalized occupancy attributed to Lrp sites. Nevertheless, we see that the IPOD-HR occupancies at known Lrp sites indeed show good correlations (**Fig.** 2B) with Lrp ChIP-seq occupancy, and that in particular, the correlation of the IPOD-HR profile with Lrp ChIP-seq at Lrp sites is substantially higher than that observed at the sites of any other global regulator. (We expect that similarly good correlations would be observed between the IPOD-HR profiles and the ChIP-seq of any of those regulators, if performed under equivalent conditions and evaluated only at the known sites for the regulator under study).

***Comment:***

*Authors may achieve this goal by genome-wide scanning of the de novo motifs (matched with known TF binding motifs) in the occupied chromosomal regions or using similar approaches. This type of analysis is necessary to assess the full potential of their technology and to uncover the conditional partition of TF regulons, which would offer key insights about the architecture and dynamic of E. coli TRN.*

**Response:**

We appreciate the reviewer’s excellent suggestions regarding these potential extensions to our inference of new likely regulatory motifs. As suggested by the author, we have performed genome-wide scans of the potential binding sites for these motifs, and provide both their likely binding sites (**Supplementary Data 4**) and an enumeration of GO terms that are enriched among the likely regulatory target of each motif (**Supplementary Data 5**). We believe that the latter item is of particular utility for identifying likely functional roles for the newly inferred motifs. Notably, we were able to identify enriched GO terms in the predicted regulons of more than 90% of the newly inferred motifs, and this result holds even after pruning any of the new motifs with potential similarity to previously known TF motifs.

***Comment:***

*Without this level of resolution, ChIP-seq experiments would still be required to compare the binding profiles of any TF of interest across multiple conditions, reducing the value of IPOD-HR. Finally, I encourage the authors to compare the IPOD-HR reconstructed TF regulons with the information available in RegulonDB.*

**Response:**

As noted in response to the previous point, we have now scanned the genome for likely binding sites for each of the newly discovered motifs, and inferred likely regulons for them (and the physiological processes implied by those regulons). We believe that deeper inference about the binding profiles of those TFs would require IPOD-HR data across a broader range of physiological conditions than the three that we have studied here (excluding genetic perturbations); we now explicitly note this in the text:

In the future, tracking of the occupancy of these motifs over a broader range of physiological conditions would likely yield additional insight into the likely stimuli sensed by them, and likely downstream processes that they regulate.

More generally, pertaining to the reviewer’s point about the value of IPOD-HR relative to ChIP-seq experiments, we believe that we have demonstrated throughout this work that IPOD-HR can in fact reconstruct the condition-dependent binding profiles of individual transcription factors across conditions, so long as there is knowledge of where to look for that binding. As an additional example (also requested by reviewer #1), we selected the case of the Lrp regulon, as we have recent ChIP-seq data for Lrp under conditions similar to those employed in our IPOD-HR experiments. We would expect *a priori* that the IPOD-HR occupancy profile across conditions at known Lrp sites be correlated with Lrp ChIP-seq occupancy under the same conditions, although the correlation should by no means be expected to be perfect, both because binding of other factors may overlap Lrp sites, and because changes in binding of non-Lrp factors may impact the normalized occupancy attributed to Lrp sites. Nevertheless, we see that the IPOD-HR occupancies at known Lrp sites indeed show quantitative correlations with Lrp ChIP-seq occupancy (**Fig. 3B**), and that in particular, the correlation of the IPOD-HR profile with Lrp ChIP-seq at Lrp sites is substantially higher than that observed at the sites of any other global regulator. We expect that similarly good correlations would be observed between the IPOD-HR profiles and the ChIP-seq of any of those regulators, if performed under equivalent conditions and evaluated only at the known sites for the regulator under study. In the context of these findings, the key value of the newly inferred motifs in particular is that they provide us with sites of interest to investigate, and to track the occupancy of, across conditions; IPOD-HR data can then be used as an effective way to perform multiplexed tracking of occupancy at many classes of sites across conditions, once the sites themselves are defined.

We address the particular comment regarding comparison with data in RegulonDB below, in response to the reviewer’s comments on lines 360-365 and 397-406.

***Specific comments***

***Comment:***

*-L141-148: the authors should clearly explain in 2-3 sentences the main changes in IPOD-HR with respect to IPOD (RNA polymerase occupancy correction, etc). What are the main changes in the analytical framework? It is unclear what the authors meant.*

**Response:**

We now provide an expanded description of the key changes in IPOD-HR relative to the original IPOD method, at the indicated place in the text (reproduced below):

On the experimental end, IPOD-HR incorporates a more stringent washing procedure (the inclusion of a Tris base wash and an additional interphase extraction step to increase the specificity of isolation of protein-DNA complexes), as well as pretreatment of the cells with rifampicin prior to crosslinking to minimize the contributions of RNA polymerase occupancy to the observed signal, and includes an RNA polymerase ChIP-seq experiment in parallel with the total protein occupancy profiling in order to allow separation of RNA polymerase occupancy from that of transcriptional regulators and nucleoid associated proteins. On the computational end, IPOD-HR makes use of a completely re-written analysis pipelines (as detailed in Methods) that provides proper adjustment for RNA polymerase occupancy, and gives an integrated workflow for calculation of total protein occupancy (including uncertainty estimates) and for the identification of key occupancy features such as occupancy peaks corresponding to individual binding sites, and large regions of high protein occupancy that act as silencing complexes on the *E. coli* genome.

***Comment:***

*-Fig 2E: authors should evaluate the statistical significance of IPOD-HR occupancy.*

**Response:**

As requested, we have added both descriptive statistics and permutation tests to more quantitatively assess the differences between the populations in **Figure 2E**; all are shown in the newly added **Table S1**.

***Comment:***

*-Fig 3B: do the TF modules indicate co-regulation? In other words, do TFs that cluster together share target genes or they only have similar average occupancy profiles?*

*Moreover, do the authors use any TF regulon size threshold when computing average occupancy score? If not, how do the authors correct for overlap between regulons (i.e. between small regulons and large regulons)?*

**Response:**

In order to address the first question, that of whether TF modules indicate co-regulation, we calculated the Jaccard indices of the gene sets regulated by co-clustered TFs vs. other pairs. We found no evidence for co-clustering indicating co-regulation; thus, it appears that TFs with similar condition-dependent occupancy profiles act independently of each other. These findings are reported in the added text noted below. Similar conclusions are reached if one tests the average Jaccard index of within- vs between-cluster TF pairs instead of the probability of having a non-zero overlap.

In order to test whether the co-clustering more generally indicated a significant overlap in the regulons of factors, or merely that they respond similarly to changing conditions, we calculated the pairwise Jaccard indices (that is, the size of the intersection of regulons between two TFs divided by the size of their unions, in terms of number of regulated genes) for all pairs of TFs considered in our analysis. We then compared the distribution of Jaccard indices for TFs which came from the same cluster, to that of Jaccard indices for pairs of TFs that did not co-cluster in **Fig 3C.** TF pairs that co-clustered were almost equally likely to have a non-zero Jaccard index (and thus share at least some regulatory targets) than were TF pairs from different clusters (odds ratio 0.95, p=0.76, Fisher’s exact test). Thus, co-clustering appears to reflect different transcription factors that have similar condition-dependent occupancy profiles, rather than factors that regulate identical targets (likely minimizing redundancy of regulatory information in favor of combinatorial regulation of a single target by factors that each sense different stimuli). We expect that substantially more insight into these patterns may be obtained in the future through application of IPOD-HR to a broader array of conditions.

Regarding the second question, that of whether a TF regulon size is used in computing average occupancy scores, we would like to clarify that the clustering is being done based on average site-level protein occupancies for the annotated binding sites of each factor under each condition. This corrects for the factors identified by the reviewer in two respects:

First, no matter how many sites are annotated for any particular factor, it will still have a single (averaged) score per condition, and thus the clustering is being performed on vectors of equal sizes. The occupancy scores of TFs with fewer annotated sites will necessarily be noisier, and we removed from consideration any factors with fewer than three detectable sites, but in general the same number of scores will be present for each TF. Second, because we focus specifically on the site level occupancies (which usually apply to distinct chromosomal locations even if two TFs regulate the same gene), there is nothing inherently problematic with comparing a large and a small regulon; the occupancies of their sites will be independently calculated and then compared. That this issue of regulon sizes does not pose a problem can be seen from the fact that TFs with large, well-characterized regulons are fairly well distributed in the clustering in **Figure 3B**, but often co-cluster (for example PhoB with ArcA, CRP with H-NS, ArgR with IHF, all of which have dozens to hundreds of sites in our analysis), and yet also often co-cluster with TFs with smaller regulons.

***Comment:***

*-L331-333 & Supp. Data S1: the authors, if possible, should include information about TFs potentially binding to the identified peaks. This information will increase the value of the data generated in this study.*

**Response:**

We agree that such information would be very useful, and had hoped to provide possible assignments by cross-referencing our peak locations with the TEC database of genomic SELEX data to identify factors that might bind each location. Unfortunately, the TEC maintainers have not replied to multiple emails to their primary contact address requesting the raw occupancy contained in their database, preventing us from performing these comparisons in an automated way. We believe that the peak sets contained here are still highly useful in several scenarios, however: (i) peaks located in a region of interest could easily be manually cross-referenced with TEC or other datasets to provide potential peak identification; (ii) any new ChIP or related experiments showing binding at these peaks may be able to use our data to immediately obtain the condition-dependent occupancy of that location; (iii) our peak locations could easily be cross-referenced with any computational predictions of TF binding sites (based on existing motifs) to provide potential assignments. We also enumerate these possible applications through the following addition to the text:

This extensive map of chromosomal occupancy and its dynamics provides the community with a wealth of known and putative novel regulatory interactions that can be further explored and validated by follow-up experiments such as those shown in **Fig. 4.** It is also possible to cross reference the newly identified binding sites with high throughput studies of potential binding locations for newly characterized TFs (e.g., ChIP or genomic SELEX (Shimada et al. 2018) data) to identify potential factors binding to a region of interest, and to use the IPOD-HR occupancy maps to obtain initial estimates of the condition-dependent occupancy of any identified sites.

***Comment:***

*-L360-365: an important piece of information missing in the current version of the manuscript is the recall rate for known TF regulons (i.e. how many of the known targets of each TF was identified by IPOD-HR). This is information is important to validate your results beyond TF motifs. Additionally, did the IPOD-HR data discover novel targets for previously characterized TFs? If yes, the authors should describe these findings. Finally, can the authors evaluate if the reconstructed regulons are supported by transcriptional data (collected in this study) or publicly available data? This would clarify the function of the putative binding events.*

**Response:**

We greatly appreciate the many excellent suggestions given here for ongoing analysis and experiments, many of which will surely prove to be promising avenues of future investigation. Unfortunately, several technical hurdles preclude the precise sort of analysis suggested here by the reviewer using the data currently in hand. While information is requested on the recall rates for known TF regulons, we must note that the regulons currently present in the literature are assembled from many datasets over many conditions. While several physiological conditions have been approached in the present study, we are nowhere near coverage of the full range of conditions that would need to be studied to expect observation of binding at most or all currently annotated sites. Compounding this challenge, several of the (excellent) suggestions here rely on the notion of reconstructing TF regulons based on the IPOD-HR data. At present, however, because we cannot confidently assign what TF is causing any particular occupancy peak, we cannot speak of reconstructing regulons. Likewise, questions such as “*did the IPOD-HR data discover novel targets for previously characterized TFs*” cannot be answered because while we identified many novel peaks, we cannot say with confidence what factor is binding to each one.

In the longer term, we expect that all of the above challenges will be met by the same two solutions: First, the gathering of IPOD-HR data under several additional physiological conditions (which we are currently pursuing), and second, the development of a computational method allowing deconvolution of the occupancy signal into contributions from specific DNA binding proteins based on principles of co-variation in occupancy of sites for the same factor across conditions (which we are currently pursuing). Thus, while all of the items listed here are worthy long-term goals, they are not yet technically feasible. We have focused our initial spread of conditions on variations in nitrogen availability, which permitted us to provide strong validation of our methods for the cases of well-studied TFs such as ArgR, PurR, and others (**Figs. 3A,3B,** and **3D**, for example). Ongoing applications of IPOD-HR and corresponding advances in our postprocessing approaches will permit many of the excellent aims suggested by the reviewer here to be accomplished in the near future, but they are in our opinion beyond the scope of the current work.

***Comment:***

*-L397-406: the authors should evaluate the overlap between regulons associated with newly inferred motifs and previously characterized TF regulons. This comparison may offer information about the potential role of the uncharacterized TFs and combinatorial regulation.*

**Response:**

We thank the reviewer for this excellent suggestion. We have applied the proposed analysis in detail to the motifs shown in **Figure 5F**, and also to the entire set of non-redundant newly inferred motifs that we report. In general, the trend that we observe is that while overlaps sometimes exist between a new motif and the regulons of one or more well-characterized factors, the overlaps tend to be limited, consistent with the newly inferred motifs having distinct regulons that often overlap those of several well-characterized TFs. We describe our application of this method in the newly added text below, and provide the full overlap statistics in **Supplementary Data S8**.

In order to provide additional insight into the potential physiological roles of the factors binding these motifs, we considered the similarity of their putative regulons to those of well-characterized transcription factors, making use of the Jaccard index comparing the gene sets potentially regulated by each motif with those of known TFs. For the top motif shown in **Fig. 5F**, the best match is for CRP, with a Jaccard index of 0.19. This finding is consistent with the putative role in regulating carbon source utilization, although the regulon of the new motif is clearly distinct from those of any well-characterized factor given that the highest Jaccard index observed for it was 0.19, and the next highest hit after CRP was 0.12. Applying the same analysis to the bottom motif shown in **Figure 5F** shows that the strongest detectable similarity in regulons is to those of H-NS (0.12) and Fur (0.11). These matches indicate potential roles for the new motif in coordinating responses to changes in iron starvation and other stress conditions such as changing temperatures, but again, the regulon of the newly identified motif is clearly quite distinct from that of the characterized TF. In order to facilitate similar analysis of the potential functions of the other newly inferred motifs, we provide in **Supplementary Data S8** the Jaccard indices for all significant overlaps between the putative regulons of our newly inferred motifs, and those of existing factors as annotated in RegulonDB (see caption for details).

***Comment:***

*-L530-534: It is unclear to me how you would compensate for the lack of prior information when using the IPOD-HR technology for reconstructing TRNs of uncharacterized species?*

**Response:**

While IPOD-HR cannot by itself provide a complete reconstruction of a new TRN, it can provide highly useful information in the form of likely binding sites (and their condition-dependent occupancy), the signs of regulatory output of those sites (though the inclusion of RNA polymerase occupancy data), and likely regulatory sequence motifs that give rise to those sites. We provide further detail on these contributions, while at the same time clarifying what the method cannot provide on its own, in the following additions to our Discussion text:

Furthermore, the ability to identify likely regulatory sites even in the absence of prior knowledge, as shown both for isolated promoters (**Fig. 4**) and inference of entire regulons (**Fig. 5**), will substantially accelerate our ability to complete a wiring diagram for the *E. coli* transcriptional regulatory network and to rapidly approach the networks of other less well-characterized bacterial species. In the latter case, we expect that the ability of IPOD-HR to highlight the locations of transcription factor binding sites, and the demonstrated feasibility of inferring DNA-binding protein sequence motifs based on those sites, will allow the rapid identification of sites of interest for detailed follow-up studies (e.g., application of pulldowns similar to those in **Fig. 4** to identify the precise factor(s) occupying a particular motif), providing an essential set of building blocks to aid in the overall reconstruction of a transcriptional regulatory network. In addition, tracking of the occupancy of different motifs across conditions will likely assist in the identification of biological conditions pertinent to the function of the factor binding each motif, and cross-referencing with RNA polymerase occupancy will provide evidence on the sign of regulatory output associated with each site. IPOD-HR thus provides a powerful high-throughput *in vivo* approach tracking occupancy at native sites, complementing methods based on screening with purified proteins [[23]](https://paperpile.com/c/E7umDe/RH3y), computational inference [[25]](https://paperpile.com/c/E7umDe/E65i), or reporter assays [[24]](https://paperpile.com/c/E7umDe/F1ze).

***Minor comments:***

***Comment:***

*-L60-62: and not all binding events are functional. Especially in the case of TF over-expression commonly used in ChIP-seq experiments.*

**Response:**

We appreciate and agree with the reviewer’s comments here, and we have added a parenthetical note to bring it to the reader’s attention:

The authors attribute this partly to the fact that even when a TF is expressed, in many cases it will not bind its targets in the absence of additional signals [[8]](https://paperpile.com/c/NGxbzd/0BeQV) (although it is quite possible that confounding effects due to TF binding sites that are not regulatory sites [[9]](https://paperpile.com/c/NGxbzd/z56BA), or experimental artifacts due to TF overexpression or epitope tagging, may also have contributed).

***Comment:***

*-Fig 3A: authors should indicate how many target genes were used for estimating the occupancy of each TF.*

**Response:**

We have added this information to the figure caption.

***Comment:***

*-Fig 3C: please add the actual correlation score. Is this anti-correlation a general trend (if you choose any random genes) or specific for the ArgR and PurR targets?*

**Response:**

We appreciate the reviewer’s comment on this point, but we believe that in fact the precise correlation at the site level is not the pertinent comparison. Admittedly we inadvertently raised this consideration through our use of the term anti-correlation, but in fact our purpose was not to compare the correlations in the individual site-level responses, but rather, simply to point out that the overall behavior across the sites shows a loss of protein occupancy at repressive ArgR and PurR sites, and a corresponding increase in transcription of their targets. We have modified the wording in both the main text and figure caption to avoid the term ‘anti-correlation’ and more precisely note the key occupancy changes being shown.

The reviewer may nevertheless be interested to know that for the comparisons shown in this figure, the Spearman correlations are -0.56 for the annotated repressive ArgR sites and -0.60 for the PurR sites, as would be expected for the occupancy of repressor binding sites. In the same comparison annotated activating ArgR sites (not shown in Fig. 3D) show a Spearman correlation of +0.65 between changes in occupancy and changes in expression of their targets. However, we do not think that site-level comparison between strength of changes in occupancy and regulatory output for any particular factor is a very useful comparison, due to the necessarily different set of regulatory inputs being integrated at each target promoter, which would confound any such comparison.

***Comment:***

*-L764: please remind the reader why rifampicin is used.*

**Response:**

We have added the following note at this point in the text to clarify:

The remainder of the culture was treated with rifampicin to a final concentration of 150 µg/mL, and incubated for 10 minutes under the same culture conditions as the main growth to immobilize initiating RNA polymerase at active promoters and permit completion of transcripts in progress.

***Comment:***

*-L828: for how many conditions did the authors perform RNA polymerase ChIP-seq with samples that were not from the same culture used for IPOD? Can the authors show that using different samples for IPOD and RNApol ChIP-seq does not change the estimated TF occupancy profiles?*

**Response:**

Non-matching RNA polymerase ChIP vs IPOD samples were used in one (out of four) replicates for the WT M9/rdm/glu condition, one (out of two) replicates for the WT M9/min/glu condition, two (out of two) replicates for the ΔlexA condition, and one (out of two) replicates for the stationary phase condition. The pooling of all available data in the analysis is justified by the reproducibility of our experiments. Within any single condition, the average pairwise Spearman correlations for the read densities across the genome obtained from different biological replicates were 0.88 for IPOD samples, 0.92 for input samples, and 0.91 for ChIP samples. Furthermore, our uncertainty estimates for key quantities such as the site-level occupancies shown in **Fig. 3A** explicitly makes use of the most pessimistic possible combination of replicates for each individual data type, and thus conservatively accounts for any day-to-day variations in the measured occupancies (which, given the correlations noted above, appear to be small).

***Comment:***

*-L1142: No access to the data (GEO requires a password)*

**Response:**

We apologize for the omission of the GEO reviewer access password, which was in the cover letter of our initial submission but apparently did not propagate to reviewers. The reviewer password is gfwpeoykdrgzzwb

***Comment:***

*-Table S4: not available.*

**Response:**

Table S4 was likewise inadvertently omitted from the initial submission, and has now been included.

*Reviewer #3 (Significance (Required)):*

*See above.*

*The reviewer field of expertise is reconstruction of gene regulatory networks.*
