# Supplementary material for: Dynamic landscape of protein occupancy across the Escherichia coli chromosome
Source: PLoS Biol. 2021 Jun 25;19(6):e3001306. doi: 10.1371/journal.pbio.3001306 (PMC8282354; doi:10.1371/journal.pbio.3001306)
Supplement: Attachment — Submitted filename: ipod_response_may2021.docx [file pbio.3001306.s027.docx]

We thank the reviewer for the additional suggestions, which we agree would further strengthen the utility of our work for the community. As noted below, we have implemented all three suggestions by the reviewer. The reviewer’s comments are given in *italics*, and we describe in response how we have added material to the manuscript to address each point.

*1. As a method & resource article, would it be possible for authors to add a column with the information regarding the closest gene to each motif binding site listed on Supplementary Data 4? I imagine authors already compiled that information in order to perform the GO enrichment analysis shown in Supplementary Data 5.*

We thank the reviewer for this suggestion, which we agree certainly makes the positions of the newly identified motif binding sites easier to interpret. We have added two additional fields to Supplementary Data 4: First, the identity of the gene closest to each motif site (as suggested by the reviewer); second, the identities of any genes identified in our analysis as potentially regulated by that binding site. Because we use proximity to annotated promoters, rather than to a gene itself, as an indicator of regulatory potential (and then extend that potential to the entire annotated operon), we believe that this information is also useful in identifying likely regulatory effects.

*2. I understand the authors' response about the current limitations to reconstruct individual TF regulons using a limited set of conditions. However, I still don't understand why the same genome-wide scan of motif binding sites used to generate Supplementary Data 4  cannot be applied to the non-redundant motifs that matched with known DNA binding motifs for E. coli? This could offer potential information about individual (partially characterized) TF regulons. Authors could highlight the caveats they have mentioned so readers are aware of any limitation when interpreting the data.*

We appreciate the reviewer’s point here, and do see the potential utility of this information. To provide the requested analysis while at the same time setting up an appropriate context, we have added an extended discussion in Supplementary Text S2 and Supplementary Figure S8 where we calculate the overlaps of inferred motifs that have TOMTOM hits to actual motifs to the true regulons for the same factors, and compare the performance of the inferred motifs to the performance of established motifs for the same factors from SwissRegulon. We find that the vast majority of the inferred motifs show good overlap enrichments with the known binding sites for the factors that they seem to resemble, although the performance is (unsurprisingly) not as strong as for the directly characterized motifs for the same factors.

*Minor point:

3. Fig S2 panel labels are missing. Also, authors referred to TF clusters in their response but those are not shown in the figure S2.*

We thank the reviewer for identifying this omission. We have added panel labels and the cluster identities as requested.
